# Isolation of *Vibrio cholera* El Tor Inaba From *Lemna minor * and *Eichhornia crassipens* Roots in Veracruz, Mexico

**DOI:** 10.5812/jjm.6855

**Published:** 2014-03-01

**Authors:** Edgar Cordoba Aguilar, Marisol Herrera Rivero, Alberto Rubi, Omar Arroyo-Helguera, Rocio Coutino Rodriguez

**Affiliations:** 1 Veracruzana University, Universitary zone, Veracruz, Mexico; 2 Institute of Public Health, Veracruzana University, Veracruz, Mexico

**Keywords:** *Vibrio cholera*, Lemnan, *Eichhornia crassipens*, *Vibrionaceae*

## Abstract

**Background:**

During epidemic periods, the strain *Vibrio cholera* *El Tor* has been isolated from the aquatic macrophyte roots of *Eichhornia crassipens* and *Lemna minor*, suggesting that aquatic plants could be environmental reservoirs through either a non-specific association or a commensalism relationship. Therefore, it is important to understand *V. cholera* reservoirs in order to establish prevention strategies against this pathogen.

**Objectives:**

Our interest was to determine whether *V. cholera* could be isolated and typified from *L. minor* and *E. crassipens* roots.

**Materials and Methods:**

From 2004 to 2005, plants were collected from various ecological niches and the roots were used to isolate *V. cholera.* Standard bacteriological, biochemical and serological tests were used for its typification.

**Results:**

In five out of the nine ecological niches explored, we collected either *L. minor* or *E. crassipens*, as these specimens cohabited only in two niches. *V. cholera* was isolated from both *L. minor* and *E. crassipens* roots. The isolated *V. cholera* showed the same biochemical characteristics as the pure *V. cholera* strain which was used as a control. The isolated *V. cholera* corresponded to *V. cholera* O1 El Tor Inaba, which is the same serotype related to the last outbreak in Mexico.

**Conclusions:**

For first time *V. cholera* El Tor Inaba has been isolated several years after the last emergence of cholera in Mexico. A viable and cultivable *V. cholera* strain, sourced from freshwater niches in *E. crassipens* and *L. minor* roots, suggests the importance of these plants as a permanent aquatic reservoir for these organisms. The monitoring of *E. crassipens* and *L. minor* is the responsibility of health institutions in order to evaluate the ongoing risks.

## 1. Background

Cholera is epidemiologically interesting for two reasons. First, it produces a severe life-threatening secretory diarrhea, frequently accompanied by vomiting, which may lead to hypovolemic shock and acidosis. In cases of delayed treatment it may lead to death, moreover cholera appears in some places causing true pandemics (1, 2). Second, cholera is endemic and epidemic in areas with poor sanitation. It is caused by certain strains of *Vibrio cholera *belonging to the *Vibrionaceae* family, Gram-negative Enterobacteriaceae. Until 1991 to 1992, cholera was believed to be caused by only two serotypes: Inaba and Ogawa, and two biotypes: classical and El Tor. All of them belong to the toxigenic O1 group with enterotoxin activity (2-4).

The origins of cholera have been elusive, but it clearly shows that it is a marine borne disease. However, standard bacteriological procedures for isolating *V. cholera *from environmental samples between epidemic intervals have not been successful. This suggests that when conditions are unfavorable for growth, *V. cholera* could enter into a dormant stage, which is viable, but not cultivable and it could be maintained in reservoirs (1). The association of *V. cholera* with plankton, as copepods and phytoplankton, is considered to be evidence, not only for its marine-estuarine environmental origins, but also for the possibility that its reservoirs are to be found in the previously mentioned environment and the organisms that inhabit it. This could also explain the erratic occurrence of cholera epidemics, mainly after the rainy season, in places where cholera is endemic (1-5). However, *V. cholera* El Tor has also been isolated from *Lemna minor *(*L. minor*) and *Eichhornia crassipens* roots, in the intervals between epidemics (1-3, 6), which suggests that *V. cholera* can live in freshwater environments, where it is generally associated with solid surfaces like crustaceans and probably some aquatic plants such as *E. crassipens* and *L. minor*. Hence we were interested in isolating and typify *V. cholera* from *L. minor* and *E. crassipens,* after the last sanitary emergency in Mexico, in plants collected in 2004 and 2005.

## 2. Objectives

The principal goal of this study was to investigate whether *V. cholera* could be isolated and typified from *L. minor* and *E. crassipens* roots.

## 3. Materials and Methods

### 3.1. Collection of Lemna minor and E. crassipens

In this study ten habitats were studied in the state of Veracruz, Mexico.

1. Casablanca Lagoon, Xalapa,
2. El Castillo Lagoon, Xalapa,
3. road between La Gloria and Zapotito, Ursulo Galvan,
4. road between Jareros and Los Idolos, Actopan,
5. road from San Juan to Vargas, Veracruz,
6. Nevaria, old national highway, Xalapa,
7. 3 km of the Veracruz-Cardel Highway,
8. 5 km before La Mancha ecological reserve, beside the Cardel-Laguna Verde Highway,
9. 31 km of the Cardel-Nautla Road, and
10. the Ursulo Galvan River.

The plants were collected in sterile bags and rinsed with a chloride solution between 2004 and 2005. Water was also collected in sterile flasks to be tested for *V. cholera*.

### 3.2. Isolation and Typification of *V. cholera* From Roots

The roots were stained with Gram solution and orange acridine at 2% in 50 mM of sodium acetate pH 3.5 and examined under a microscope using 40X and 100X objectives. After two to five days, the roots of both plants were grown either directly in thiosulphate citrate bile salts (TCBS), or in alkaline peptonate water at pH 8.5 (APW), with 1% isotonic sodium chloride media, and incubated for approximately 18 and 24 hours. In order to eliminate other vibrions, the cultures were previously incubated at 42ºC for 8 hours. Cultures in APW were Gram stained and later spread on Salmonella Shigella agar (SS) and thiosulfate, citrate and bile salts (TCBS). In TCBS, the colonies were typified using standard biochemical tests.

### 3.3. Hemolysin Activity of Group O

Healthy human erythrocytes from group O blood were washed 2 or 3 times with Dulbecco’s phosphate-buffered saline, lot AMB 15477 (PBS), to prepare a 2% bacterial culture solution of *V. cholera* isolated from *E. crassipens* and *L. minor* (pure cultures from selective media) grown in non-selective media (nutritive broth) for a period of 24 to 48 hours. After centrifugation at 2000 rpm (Sorvall Super T 21 rotor Sorvall SL 50 T), the supernatant was used as a source of hemolysin. A supernatant was placed in a 96-well microplate containing 2% erythrocyte solution. Double serial dilutions were performed, and the hemolytic titer for both cultures was obtained. In addition, 2% lamb blood agar was used in order to determine hemolysin activity. The lamb’s blood was obtained from the slaughterhouse, the erythrocytes were washed twice with a PBS solution, and a 2% solution of lamb blood erythrocytes was prepared and mixed with agar and placed in Petri dishes. Then 2% bacteria solution of *V. cholera* either *L. minor* or *E. crassipens* grown in non-selective media was spread and incubated at 37°C for 18 hours; afterwards a hemolysin ring was detected giving a positive test result.

### 3.4. Serum Typification of Vibrio cholera

Once *V. cholera* was typified from *E. crassipens* and *L. minor, it* was isolated in the TCBS culture, and sown in non-selective media, such as agar infusion or tripticase soy agar, and incubated at 37°C for 18 to 24 hours. After gathering several smooth colonies, they were resuspended or emulsified directly in sodium chloride at 0.85%, or in a PBS solution to create a bacterial solution. Thereafter, a drop of bacterial suspension was placed on a plate or microplate, and a drop of tested antiserum was added. Lastly, the agglutination was determined visually, or through changes in the OD values at 560 nM. The antiserum dilution was made as previously indicated. To identify the biotype and serotype of *V. cholera,* we used polyvalent and monovalent antiserums for the *V. cholera* O1 agglutination test, with polyvalent antiserum for *cholera* lot 57142 (Sanofi Diagnostics, Pasteur 92430 Marmens, la Coquete, France) and polyvalent 01, number INI 911, and monovalent Ogawa INI 912 and INABA INI 913 (Interbiol Carretera Federal Mexico-Pachuca, Km 484, Zona Industrial, Mexico, D.F). In addition, capsular polyvalent antiserum for Shigella lot SH2-06-04 (BioRad) and for Salmonella polyvalent S1 cat 110504 (SANOFI) were used as negative controls.

### 3.5. Vibrio cholera Immune Fluorescent Typification on Roots

The same primary antibodies were used for serum typification (polyvalent O1 antibodies for *V. cholera*, S1 for Salmonella, SH2 for Shigella, monovalent antibodies for Ogawa, and Inaba for *V. cholera*). The roots were placed in cold acetone for 45 minutes at 4°C. They were washed twice with PBS and thereafter, per primary antibody, two roots were placed in duplicate in each well of the microplate. The OD was determined at 405 nM. Subsequently, they were incubated with PBS and respective primary antibodies (polyvalent and monovalent antiserums) for 4 hours at room temperature. The roots in the PBS were used as a negative control. Then they were washed twice with PBS, and again the OD was determined at 405 nM. Subsequently, the PBS were added, along with fluorescent secondary antibodies, and again incubated for 4 hours or overnight, then washed twice with PBS to determine the OD at 405 nM. The dilution of the secondary antibody was 1:800 in order to reduce background stain. As the antibodies are very specific, we expected to detect an increase in the OD values in positive cases after the addition of the primary and secondary antibodies.

### 3.6. Statistical Analysis

Differences between the groups were assessed by Student´s t-test. P values < 0.05 were considered as statistically significant.

## 4. Results

**Figure 1.**
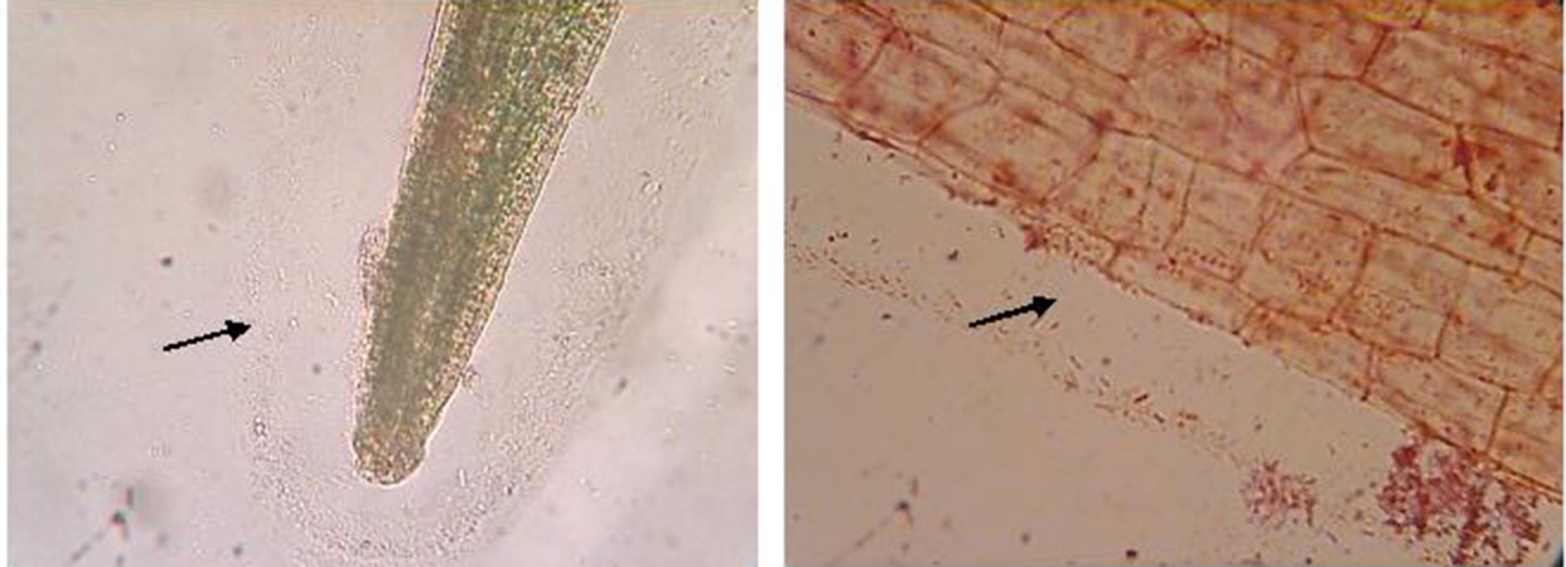
Pictures From Gram-Stained *L. minor* Roots Under a Nikon Microscope Using 40X and 100X Objectives Arrow shows a biofilm or thin layer around the roots with the presence of Gram-negative Bacilli

Collection of the plants and isolation of *V. cholera* from the plant’s roots were conducted in nine different niches in Veracruz. Samples of *L. minor* and *E. crassipens* were collected from five areas and only in two of those niches were they found together. One was located in the Ursulo Galvan River, and the other 31 km along the road from Cardel to Nautla. Taxonomic identity was confirmed by the herbarium of the National Institute of Ecology (Veracruz, Mexico). Gram-negative bacteria were detected, principally adhering to a clear thin layer of mucin biofilm around *L. minor* roots, as shown in Figure 1. 

In the samples, *V. cholera* was isolated from either *L. minor* or *E. crassipens*, and biochemically typified from their roots. After being cultured in APW and spread or directly sown on TCBS, they showed colony morphology, similar to *V. cholera*. Colonies grown in TCBS from *V. cholera* isolated from both *E. crassipens* and *L. minor* were very similar to the control *V. cholera* pure strain, as shown in Table 1. However, some colonies of the *V. cholera* obtained from *E. crassipens* possessed a different morphology (being yellow, flattened and lobulated, with an amoeboid aspect such as in the rugose colonies). They also required more time to develop than the ones from *L. minor* in TCBS.

**Table 1.** *Vibrio cholera* Colonies Morphology Isolated From *E. crassipens*, *Lemna minor* and Pure Strain Microorganisms in Thiosulfate, Citrate and Bile Salts Medium

Bacteria	Optical Characteristics	Size, mm	Form	Edge	Elevation	Chromogenesis
***V. cholera*****(pure strain)**	shiny opaque	2 - 4	dot	whole	elevated	yellow fluorescent
***L. minor*** ** root**	shiny opaque	2 - 4	dot	whole	elevated	yellow fluorescent
***E. crassipens root***	shiny opaque	2 - 4	dot and amoeboid	lobulated	elevated and some flat	yellow fluorescent and some yellow

In the biochemical tests used for identifying *V. cholera* and its biotype, all tests for *V. cholera* El Tor were positive, including; H_2_S (-), Indol (+), mobility (+), LIA (+), TSI K/A and citrate (-), oxidase (+), gelatinase hydrolase (+), and reduction of nitrate (+), as shown in Table 2. They were also compared with a *V. cholera* control strain, which showed the same biochemical characteristics.

**Table 2.** Biochemical Tests to Typify Isolated *Vibrio cholera* and Compared with the Pure Strain *Vibrio cholera* Used as a Positive Control

Biochemical Tests	*V. cholera* Pure Strain	*V. cholera* Isolated From *L. minor*	*V. cholera* Isolated From *E. crassipens*
**LIA ** ^**a**^	+	+	+
**TSI ** ^**a**^	A/A	A/A	A/A
**KIA ** ^**a**^	K/A	K/A	K/A
**Citrate**	+	+	+
**Gelatin**	+	+	+
**Oxidase**	+	+	+
**Hemolysin**	+	+	+
**SS ** ^**a**^ ** agar**			
**Indol**	+	+	+
**Motility**	+	+	+

^a^ Abbreviations: LIA, lysine iron agar; TSI, triple agar iron; KIA, Kliger iron agar; Salmonella-shigella agar.

As shown in Table 3, the isolated bacterial strains were analyzed using serological tests, such as polyvalent and monovalent Inaba and Ogawa antiserum (Sanofi and Interbol). We corroborated that the *V. cholera* strains isolated from *E. crassipens* and *L. minor* roots were *V. cholera* O1 El Tor Inaba, because we observed the agglutination caused by the O1 and Inaba antiserum, however, no agglutination was shown with the Ogawa antiserum. Finally, an immunocytochemistry technique was used in order to substantiate the presence of *V. cholera* in the roots of *L. minor* and *E. crassipens*.

**Table 3.** Serological Tests used for Typifying *Vibrio cholera*

Bacteria	Polyvalent O1	Monovalent Anti-Inaba	Monovalent Anti-Ogawa	Saline PBS
***V. cholera*** ** pure strain**	0.120	0.076	0.048	0.048
			0.080 ^a^	
***V. cholera*** ** from ** ***L. minor***	0.069	0.071	0.052	0049
			0.097 ^a^	
***V. cholera*** ** from ** ***E. crassipens***	0.073	0.130	0.05	0.048
			0.071 ^a^	

^a^ Three days later, only the Ogawa values had changed.

^b^ *Vibrio cholera* pure strain was used as a positive control, and saline as an agglutination control. The OD at 560 nm was read immediately after the test.

Finally, as shown in Table 4, a significant increase in the absorption at 405 nm was observed, compared to the control. This increase was observed after adding the first antibiotic to almost all of the antiserums, either in *L. minor* or *E. crassipens,* however, a higher increase was observed in the *L. minor* roots treated with Inaba antiserum and in *E. crassipens* roots with Ogawa. An increase was also observed in *E. crassipens* and *L. minor* roots treated with Salmonella and Shigella antiserums, respectively (Table 4). 

including three deaths, were reported between 1991 and 1997 (3). Following that date, no further cases have been reported. This means that they appear abruptly and then disappear, and again the question is always the same: What happens to *V. cholera* during those intervals when it is not detected? Most researchers believe that it remains latent in reservoirs (6-9), and it has also been suggested that these may sustain *V. cholera* for long periods of time (6-11). The reservoirs or sites of survival and multiplication of pathogenic vibrions between epidemics are not well known. It is well documented that during epidemics, toxigenic *V. cholera* O1 can be isolated from local freshwater (10, 11), but it disappears after the epidemic subsides (4, 7), which is one reason to give importance to the identification of these reservoirs. After working in vitro, previous reports have suggested that aquatic plants such as *L. minor* and *E. crassipens* are *V. cholera* reservoirs (6-9), and our results corroborate these findings.

**Table 4.** Determination of *V. cholera* from *L. minor* and *E. crassipens* Roots by Immune Fluorescent

Antibody/Roots	Saline Control	Anti O1	Inaba	Ogawa	Poly O Salmonella	Capsular Shigella
***L. minor***** primary Ab**	0.318 ^a^	0.549 ^a^	0.513 ^a^	0.578 ^a^	0.542 ^a^	0.453 ^a^
***L. minor***** secondary Ab**	0.300	0 .670	0.73	0.664	0.589	0.453
**OD secondary/primary**	0.94	1.175	1.423	1.148	1.08	1.0
***E. crassipens***** primary Ab**	0.304	0 .443	0.266 ^a^	0.696 ^a^	0.596	0.427 ^a^
***E. crassipens***** secondary Ab**	0.336	0.458	0.363 ^a^	0.925 ^a^	0.669	0.361
**OD secondary/primary**	0.9	0.96	0.73	0.75	0.89	1.18

^a^ P < 0.05 compared to control.

## 5. Discussion

Throughout history various worldwide cholera pandemics have ocurred. In Mexico, seven states have been affected, and one of those was Veracruz. In the 5th Sanitary Jurisdiction, where this study was performed, 216 cases, The presence of *E. crassipens* and *L. minor* also coincide with municipal wastes and this may indicate water contamination by feces. As those plants have the capacity of denitrification (12), they may be considered as indicators of fecal contamination and a potential risk for cholera disease. Our results, determined after biochemical and serological typification, demonstrated and corroborated the permanence and viability of *V. cholera* biotype El Tor in aquatic plants, several years after the outbreaks (7, 13).

The isolated and typified *V. cholera* El Tor, collected from either *L. minor* or *E. crassipens* roots, showed the same biochemical characteristics as the *V. cholera* isolated from a patient with cholera. We believe that this microorganism is toxigenic, considering the results obtained with polyvalent and monovalent antiserums to test *V. cholera* O1 agglutination, and its hemolysin activity in human group O and lamb’s blood. 

Using serological tests, both strains were identified as Inaba. However, we do not know if we also started with Ogawa which mutated to Inaba, as this frequently occurs (14). On the other hand we could have dealt with both serotypes, since the strains were isolated after the roots had been washed with chloride solution, which indicates high resistant capacity, as reported for the Ogawa rugose TSI-4/R strain (15), and also with the colonies morphology. According to the appearance of the *V. cholera* colonial morphologies, two-phase variants, including smooth and rugose, have been described for Ogawa. In addition, the reversible phase variation between the rugose and smooth colony variants, and associated phenotypes, is postulated to be an important factor for the survival of the organism, as it is also dependent on the nutritional conditions (15, 16). These could happen in the Inaba strain too; in any case both of these strains are known to be a public health risk.

Therefore, it is very important to conduct future studies to find out more about this association and confirm the presence of dangerous microorganisms in those plants. In addition it is also important to verify if these plants are permanent reservoirs of *V. cholera,* because this may explain some epidemiological cases, including reported cases in Peru, Hurricane Katrina and the situation in endemic zones (1, 3). Poor sanitary conditions and the fact that cholera is one of the best examples of water-borne diseases that can be controlled through the implementation of water treatment and sanitation measures, make further investigations vital. Outbreaks should be controlled with appropriate water treatment, but in affected places the residual effluent is discharged directly into the rivers where these plants grow. The presence of organic materials favors the growth of V. *cholera* roots, and this is due to stress factors such as; temperature, pH, and salinity, and they may induce the conversion from a nonpathogenic to a pathogenic phase, which is a constant risk.

Even if these plants are the only reservoirs of vibrions that are not toxigenic, they still present a high risk health, because filamentous phages live in those environmental conditions and they are involved in the transference of the toxigenic genes to *V. cholera*.
